# Optimization of the Frying Temperature and Time for Preparation of Healthy Falafel Using Air Frying Technology

**DOI:** 10.3390/foods10112567

**Published:** 2021-10-25

**Authors:** Mohammad Fikry, Ibrahim Khalifa, Rokkaya Sami, Ebtihal Khojah, Khadiga Ahmed Ismail, Mokhtar Dabbour

**Affiliations:** 1 Department of Agricultural and Biosystems Engineering, Faculty of Agriculture, Benha University, Moshtohor, Toukh 13736, Qalyoubia Governorate, Egypt; mokhtar.dabbour@fagr.bu.edu.eg; 2 Department of Food Technology, Faculty of Agriculture, Benha University, Moshtohor, Toukh 13736, Qalyoubia Governorate, Egypt; Ibrahiem.khalifa@fagr.bu.edu.eg; 3 Department of Food Science and Nutrition, College of Sciences, Taif University, P.O. Box 11099, Taif 21944, Saudi Arabia; eykhojah@tu.edu.sa; 4 Department of Clinical Laboratory Sciences, College of Applied Medical Sciences, Taif University, P.O. Box 11099, Taif 21944, Saudi Arabia; khadigaah.aa@tu.edu.sa

**Keywords:** air frying, falafel, optimization, quality properties

## Abstract

Air-frying is an innovative technique for food frying that uses hot air circulation to prepare healthy products. The objectives of this study were to establish simplified models to reflect the efficacy of the air frying process at varying temperatures and times on the quality attributes of falafel, and to optimize the frying conditions for producing air-fried falafel. Moisture content, color, fat content, hardness, and sensory evaluation of the fried falafel were analyzed under varied temperatures (140 °C, 170 °C, and 200 °C) and time periods (5 min, 10 min, and 15 min). Statistical analysis was then applied to obtain the best fit model that can describe the properties of fried falafel. Results indicated that moisture content, fat content, and L*-value of air-fried falafel were adversely related to the frying temperature and time, but the hardness and ΔE of fried falafel were increased as the frying temperature and time increased. Moreover, an increase followed by a decrease was shown for the appearance, aroma, crispness, taste, and overall preference scores with the increase in frying temperature and time. The regression analysis showed that the proposed models could be properly used for predicting the properties of the fried falafel. In addition, the overlaid plots resulted in the optimum frying temperature of 178.8 °C and time of 11.1 min. Interestingly, the fat content of the air-fried falafel reduced by 45% at optimal frying conditions compared with that for the deep-fat fried one at 180 °C for 7 min (control). In comparison, the air-fried falafel was lower in fat content, higher in hardness with more acceptable appearance and crispness scores than deep-fat fried falafel. Such information could be beneficial to the manufacturers of the falafel to produce an optimal and healthy product.

## 1. Introduction

Fried foods are popular worldwide and their development has exponentially expanded recently [1]. Meanwhile, fried-based foodstuffs are acceptable for individuals of all ages. Fried foods are well-known for their features of tasty, crunchy, and crispy [2,3]. Additionally, the beneficial nutrients such as fibers, minerals, carotenoids, etc.; freshness, and convenience of the foods are the key factors in manufacturing acceptable fried food products. For example, the “textural dichotomy of the food: dry and crispy crust, tender inside” is one of the most important reasons for the popularity of fried foods [4]. Fried foods are, in general, very high in fat content which is almost 35–44% of the total products by weight [5,6]. As a result, fats ensure high-level satiety and can cause several health risks to consumers, namely chronic non-communicable diseases [7]. Therefore, optimizing the fat content, composition, and overall quality of fried foods is critically demanded.

In this context, plenty of fried food formulas that contain vegetables as key ingredients were developed. Falafel is one of the key fried vegetables in the Middle East, especially in Egypt [8]. Falafel is a traditional Egyptian cuisine that consists of chickpeas, water, onion, garlic, spices, parsley, paprika, and sesame seeds [8]. Falafel is considered a highly nutritious product, mostly due to its vegetarian-based constitutes, such as vitamins, dietary fiber, and bioactive components [8]. Traditionally, fried falafel is produced using a deep-oil frying process which is not preferred for health-oriented individuals because of the high fat content caused by the dipping in the oil.

Commercially, fried foods can be produced using different frying techniques (e.g., deep-fat, vacuum, hot air, and stir-frying) as the frying process is an imperative unit operation that is broadly employed not only in food plants but also in eateries and homes, etc., mostly due to its easiness, fastness, and for economic aspects [9]. Different frying techniques could be utilized by food producers such as deep-fat, vacuum, hot air, and stir-frying [10]. Commonly, foods are prepared by dipping them in oils and/or fats, and this technique is known as deep-fat frying. The deep-oil frying assay is one of the eldest methods of cooking food, which is vastly valued by customers due to its taste [11]. In detail, the immersion of a product into hot oils and/or fats instigated the water’s egress and oil’s ingress and consequently induced alterations in texture and color properties of food products. Practically, it was reported that deep-fat frying has some operational-related drawbacks, including unsuitable frying duration, temperature, pressure, etc., which can be categorized such as premature foaming, premature smoking, premature darkening, “off” flavors, odors, and a typical frying performance [12]. The scientific community, consequently, is still looking for a novel technology to maintain the quality attributes of food products during the frying process, which is technically and economically suitable for food industries.

Nowadays, people are interested in low-fat/fat-free foods which could be prepared using alternative frying techniques, such as air frying technology, to overcome the limitations of deep-fat frying. The air frying method can be utilized in food cooking in which superheated air is circulated around the product instead of immersion in oil. Remarkably, air fryer device consists of a cooking chamber that can be heated by a heater and an exhaust fan which helps in providing the needed air flow to the fryer [2,9]. In this technique, gradual dehydration of the product occurs until the fried products become crusty. The existing literature indicates that air-frying considerably reduces the fat content by 80% in the final product relative to conventional frying [13]. However, many sensory properties of the product such as crust formation, palatability, color, brightness, etc., may be influenced by the lower oil content. Thus, controlling the frying process using an optimization technique to prepare fried products with high-quality characteristics, particularly fat content, composition, and overall quality, is substantially needed.

Notably, the air frying method was effectively used to cook different foods, such as fried potato [1,2,5,13,14,15,16,17], surimi [3], doughnuts [18], and fish skin [19]. Besides, the optimization tool is considered an effective technique that is used in optimizing complex processes and was extensively applied in numerous food processes [2,20,21,22,23,24]. Nevertheless, to date, no scientific reports have explored the optimum frying conditions to produce fried falafel. Therefore, the current study aimed to: (1) investigate the influence of frying temperature and time on the quality properties of the air-fried falafel, (2) establish suitable models to predict the properties of the air-fried falafel, and (3) optimize the frying conditions for preparing the air-fried falafel with high-quality attributes.

## 2. Materials and Methods

### 2.1. Air-Fried Falafel Preparation

Frozen falafel—which was prepared using the method of Ismail et al. [25], Ismail and Kucukoner [8], and Abu-Alruz [26]—was purchased from a local market in Benha, Egypt. The initial moisture content and fat content of the falafel were 55.3 ± 0.7 (% w.b.) and 16.24 ± 1.1 (% d.b.), respectively. To obtain fried falafel, the air fryer (Philips, Model HD9220/20, United Kingdom) was set at the desired temperature for 10 min at an air velocity of 4.9 m/s (air velocity value was determined by Vane anemometer, TESTO 416, TESTO, UK) to guarantee a steady-state was achieved. The frying process was conducted at three different temperatures (140 °C, 170 °C, and 200 °C) for three different times (5 min, 10 min, and 15 min). These conditions were chosen based on our preliminary study. Next, the temperature and time were adjusted at the desired frying conditions then; five falafel pieces were placed in a thin layer inside the container of the fryer without using oil. The air-fried falafel samples were collected from the fryer in less than 10 s to maintain steady-state conditions throughout sampling. All fried falafel samples were allowed to cool at room temperature (25 + 1 °C) Next, the fried falafel samples were stored in a refrigerator at 8 ± 1 °C to keep them away from deterioration until performing fat content and color analysis.

### 2.2. Measurement of the Quality Attributes of the Fried Falafel

#### 2.2.1. Measurement of Moisture Content (MC)

To determine the moisture content of the fried falafel, the procedure described by Massini et al. [27] was used. Air-fried falafel samples were dehydrated in an oven (Memmert, UN, Germany) at 100 °C until reaching a constant weight. The MC of the samples was calculated on a wet basis by subtracting the mass of the samples before and after the frying process then the result was divided by the mass of the sample before the frying process. The results were documented as the average of triple measurements.

#### 2.2.2. Evaluation of Hardness

The protocol outlined by Fikry et al. [28] was followed to quantify the hardness of the air-fried falafel. To measure hardness, an Instron Universal Testing 5566 Machine (Canton, MA, USA) was utilized. In detail, each air-fried falafel piece was placed horizontally between two flat parallel plates, then, the air-fried falafel sample was exposed to a 9.8 kg load cell at a crosshead speed of 10 mm/s following the procedure previously applied by Fang et al. [19]. The hardness was determined according to the maximum force in Newton detected from the force-deformation curve. To ensure the homogeneity of the tested sample, the sample and compression plate were perfectly aligned as it was outlined by Jonkers et al. [29]. Three runs were immediately conducted after the frying process, and the outcomes were recorded as averages of three replicates.

#### 2.2.3. Assessment of Fat Content (FC)

To determine the FC of the fried falafel, a Soxtec 2050 Auto Fat Extraction System (FOSS, Denmark) was used. Hexane was utilized as a solvent in this process. According to the method previously used by Fang et al. [19] and Abd Rahman et al. [2], the fried falafel was first dried using a lab oven (Memmert, UN, Germany) at 80 °C for 24 h. Afterward, the empty aluminum cups (dried at 105 °C for 30 min) were moved into a desiccator for cooling purposes (15 min), and then their mass was recorded using an analytical balance. Next, a mortar and pestle was used to mash and grind the fried falafel to reach the homogeneity state. The ground samples were transferred into the aluminum cups and were then submerged into boiling hexane for 40 min. The cups were dried again in the oven for 30 min and thereafter cooled for 20 min (at 25 °C). Finally, the cups with oil were weighed and the fat content (dry basis) of each sample was calculated by subtracting the initial and the final weight of the cups; then, the result was divided by the weight of the dried sample. Data were summarized as averages of triplicates.

#### 2.2.4. Colour Analysis

The color attributes L* (darkness/lightness), a* (greenness/redness), and b* (blueness/yellowness) of the air-fried falafel samples were measured using a colorimeter (HunterLab, Colour Quest^®^ XE 3399, Reston, VA, USA). The instrument was firstly standardized against white and black tiles before sample measurement. To control the changes in color throughout the frying process, the L*-value is considered a common choice due to its similarity with the color inspection made by the operator. The color attributes were determined in triplicates and the results were recorded as averages. The total color difference was calculated using Equation (1) [30]. The initial L*, a*, and b* values of falafel which were used for calculating the color difference were 68.7, 1.24, and −41.6, respectively.

(1) $$\Delta E=\sqrt{{\left(\Delta L^{*}\right)}^{2}+{\left(\Delta a^{*}\right)}^{2}+{\left(\Delta b^{*}\right)}^{2}}$$

### 2.3. Sensory Analysis of Fried Falafel

To assess the air-fried falafel samples, a sensory evaluation process was performed with 30 panelists, taking the guidelines of the standard norm into account [31]. Panelists were semi-trained consumers. They constituted persons normally familiar with the quality of falafel as they are daily consumers of this product in Egypt. Prior to performing the examination, evaluators were informed and approved to assess the fried falafel as an ethical operation, and were informed of the nature of fried falafel being evaluated and then invited to evaluate five sensory parameters of the air-fried falafel samples (appearance, aroma, taste, crispiness, and overall preference). The sensory evaluation process was conducted in a room that was environmentally controlled (25 ± 2 °C) under white fluorescent light [32,33]. According to the procedure described by Mendes et al. [34], a nine-point hedonic scale (1 = disliked extremely; 5 = neither liked nor disliked and 9 = liked extremely) was used by the panelists so that they could measure how much they liked or disliked the examined samples. Air-fried falafel samples were placed in 3-digit coded plates, which were thick, white, and fragrance-free plates, and then the samples were randomly evaluated by all panelists during three sessions. To ensure the accuracy of the sensory evaluation process, the panelists frequently cleaned their mouths using water bottles. The sensory assessment was immediately performed after the frying process, and the outputs were recorded as averages with standard deviations.

### 2.4. Comparison between Optimal Air-Fried and Deep-Fried Falafel

Optimal air-fried and deep fat-fried falafel obtained at frying conditions of 178 °C for 11 min and 180 °C for 7 min, respectively, were compared with respect to their moisture content, fat content, hardness, L*-value, and sensory attributes. To obtain deep-fat fried falafel, a deep-fat frying process was conducted using a commercial deep-fat fryer (Philips, Model HD6155, Farnborough, UK). Prior to submerging the falafel samples into cooking oil, the heating temperature (180 °C) of oil was ensured. Once the frying process ended, paper towels were used to remove the excess surface oil.

### 2.5. Design of the Experiment and Statistical Analysis

To design the experiment, a three-level two factor (3^2^) full factorial statistical design was used as presented in Table 1. To examine the effects of the independent variables (temperature, $x_{1}$ and time, $x_{2}$) on the dependent responses (moisture content, hardness, L*-value and sensory attributes), a two-way ANOVA test was performed. Furthermore, MINITAB 18 software (Minitab Inc., State College, PA, USA) was used to fit the model (Equation (2)) and to calculate the regression constants by utilizing multiple regression analysis. Furthermore, Originlab Pro 2018 software (OriginLab, Northampton, MA, USA) was used for plotting the surface and contour lines. The significance levels of all the terms in the model (Equation (2)) were statistically verified at a *p*-value of 0.05.

(2) $$y_{n}=\beta_{0}+\beta_{1}x_{1}+\beta_{2}x_{2}+\beta_{12}x_{1}x_{2}+\beta_{11}x_{1}{}^{2}+\beta_{22}x_{2}{}^{2}$$

where $y_{n}$,$\;x_{1}$, $x_{2}$ denote the responses, temperature, and time, respectively. Moreover, $\beta_{0},\beta_{1},\;\beta_{2},\;\beta_{12},\beta_{11},\beta_{22}$ are constants.

## 3. Results and Discussion

### 3.1. Physicochemical Properties of Air-Fried Falafel as Affected by Frying Conditions

**Figure 1:**
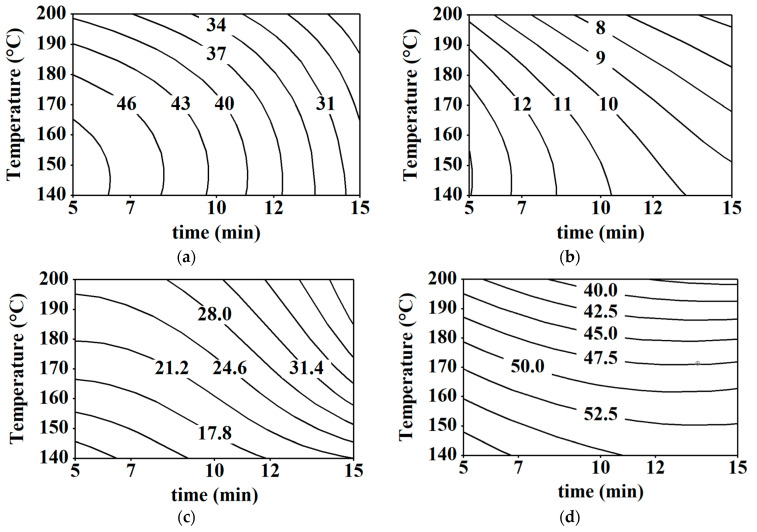

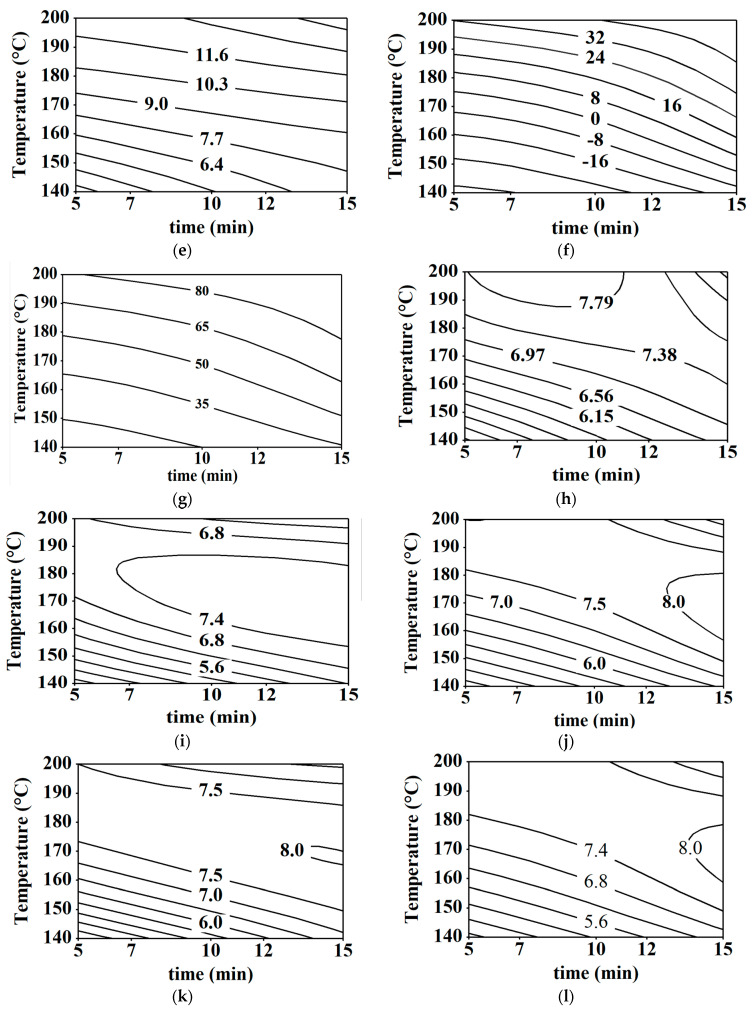
Contour plots for (**a**) moisture content (MC w.b. %), (**b**) fat content (FC %d.b.), (**c**) hardness (N), (**d**) L^*^-value, (**e**) a*-value, (**f**) b*-value, (**g**) ΔE, (**h**) appearance, (**i**) crispiness, (**j**) taste, (**k**) aroma, and (**l**) overall preference of the fried falafel at different frying conditions.

Moisture content, hardness, fat content, L*-value, a*-value, b*-value, and ΔE are important traits in the quality control of air-fried falafel. Obviously, real data of moisture content, hardness, fat content, and color attributes of the fried falafel in relation to different frying conditions (temperature and time) are plotted in Figure 1.

Moisture content in foods is essential since it is crucial for numerous quality and food safety factors [35]. From Figure 1, the moisture content values of fried falafel mostly reduced as the frying temperature and time grew. This relationship could be attributed to the dehydration of the falafel that occurred during the air-frying process. Comparable observations have also been indicated regarding the effect of temperature and time on moisture content for fried potato [2].

Hardness could be defined as the force required to reach a particular deformation. Thus, it is considered a vital indicator of the frying level and the acceptability of the food products [36,37]. It was observed from Figure 1 that the hardness of fried falafel increased as the frying temperature and time increased. In general, the hardness of air-fried falafel ranged from 8.9 and 44.6 N at different frying temperatures and times. Figure 1 shows that the lowest value of the hardness (8.9 N) was obtained at a frying temperature of 140 °C for 5 min, but the greatest value (44.6 N) was at a frying temperature of 200 °C for 15 min. Such an outcome confirmed that there was a positive relationship between the hardness and frying conditions (temperature and time). A similar trend has been found for fried potato [2]. The texture of fried products could be described by the formation of a surface crust which is the most pleasant attribute by consumers. Alterations in the exterior layers of the product at the cellular level resulted in this crusty texture [2,38].

Clearly, it can be detected that the fat content of air-fried falafel was adversely related to the frying temperature and time (Figure 1).

At the same temperature, the FC reduced as the frying time increased. A similar trend was found for fried sweet potato [2]. Furthermore, the results showed that the fat content of the air-fried falafel reduced by around 64% after being fried at maximum conditions of 200 °C for 15 min. This observation is possibly attributed to the fat drainage from the falafel while no oil was used throughout the frying process. In the air frying process, there is no oil that goes into the fried product since there is no other liquid that would replace the removed water from the pores due to capillary pressure.

**Figure 2:**
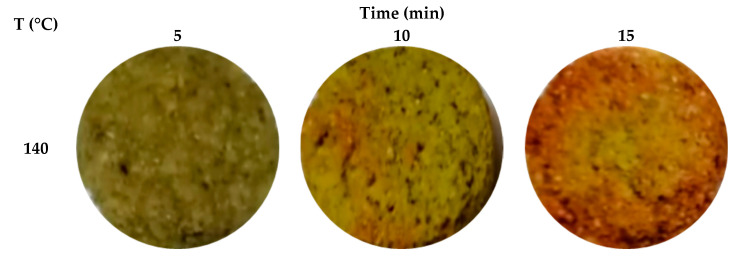

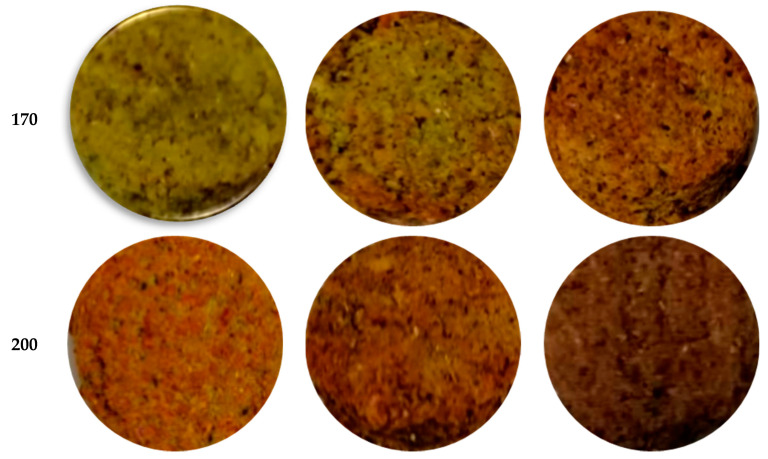
Color alterations of the air-fried falafel in relation to frying temperature and time.

On the other hand, the color attributes of fried products significantly influence consumer satisfactoriness. The lightness/darkness value (L*), redness (a*), and yellowness (b*) of the fried falafel were determined but due to L*-value is considered a vital factor in the frying industry and is commonly used as a quality control parameter, therefore it can be only focused on L*-value [39]. Usually, a dark-colored product is not preferred by consumers, thus, a suitable optimum condition is very important during the frying process to avoid food burning. Clearly, a decrease in L* values was observed with the increase in the frying temperature and time (Figure 1). Contrarily, an increase in ΔE was detected as the temperature and time increased. The observed outcome in this investigation was consistent with the previous finding realized by Abd Rahman et al. [2] for fried sweet potato. Such a finding is possibly linked to the non-enzymatic browning and pyrolysis reactions that occur during the frying process which enhance the development of brown pigments, and consequently, give the fried falafel a darker color. Heat and mass transfer during the frying process could also be a reasonable reason for physicochemical alterations that affected the color of fried foods. Figure 2 portrays the effect of different frying temperatures and times on the color of the air-fried falafel.

### 3.2. The Effect of Frying Conditions on the Sensory Attributes of the Air-Fried Falafel

The sensory properties of the fried falafel under different frying temperatures and times such as appearance, aroma, crispness, taste, and overall preference were assessed. A score equal to 6.0 on a nine-point hedonic scale could be an appropriately acceptable limit [40].

During the frying process, appearance is considered as a key quality control parameter [34,41]. Therefore, frying at a temperature of 200 °C and a frying period of 10 min showed the highest appearance score of the fried falafel (Figure 1). From Figure 1, it can be observed that the appearance score increased and then decreased as the frying temperature and time increased. These alterations in the appearance of the fried falafel may be linked with the Maillard reactions that occur during the frying process [42].

Moreover, the aroma, taste, crispiness, and overall preference are also considered vital quality factors of fried falafel. The highest aroma, taste, crispiness, and overall preference scores of the fried falafel were found to be accepted for frying conditions of 170 °C and 15 min (Figure 1), Clearly, there is an increase followed by a decrease in the aroma, taste, crispness, and overall preference scores with the increase in frying temperature and time (Figure 1).

### 3.3. Data Validation

To examine the suitability of the polynomial model for fitting the properties of the fried falafel, regression analysis was performed. Obviously, it can be seen from Table 2 that the properties (moisture content, hardness, and appearance) of fried falafel were linked to the linear, quadratic, and interaction effects of the frying temperatures and time, while there are linear and quadratic effects of frying temperatures and time of the fat content and the L*-value of the fried falafel.

**Figure 3:**
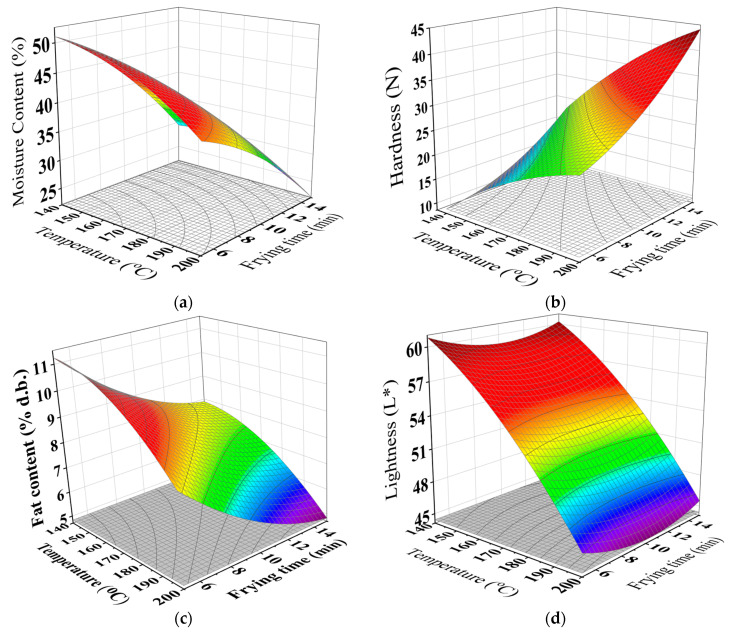

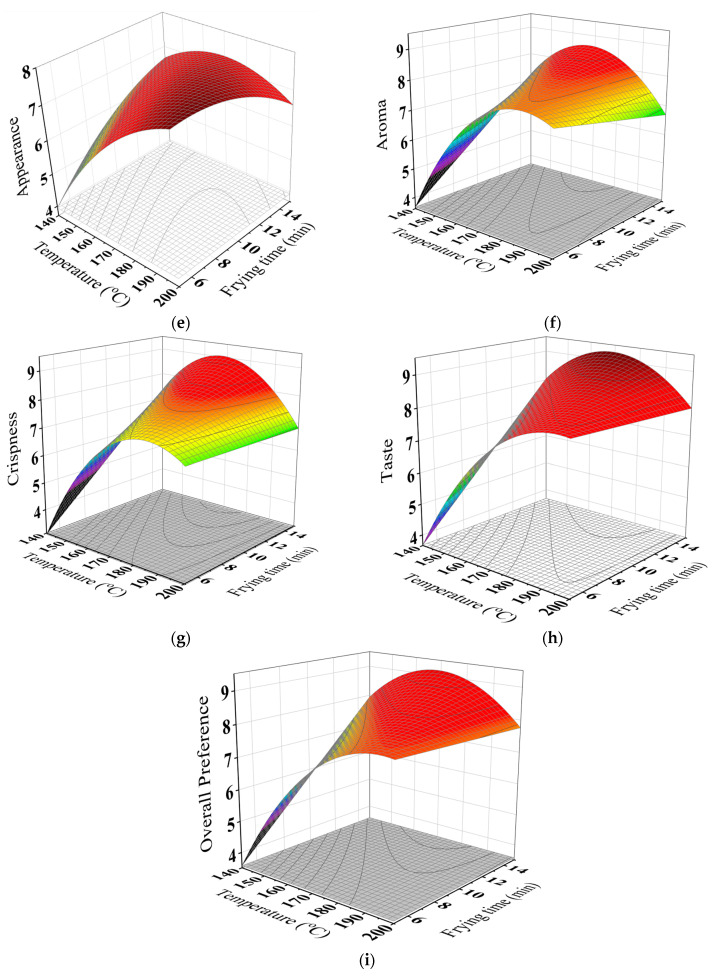
Response surface for (**a**) MC (w.b. %), (**b**) hardness (N), (**c**) FC (d.b.%), (**d**) L*-value, (**e**) appearance, (**f**) aroma, (**g**) crispiness, (**h**) taste, and (**i**) overall preference of the fried falafel at different frying conditions.

Table 2 reveals that the aroma, taste, crispness, and overall preference scores of the fried falafel were linearly associated with the frying temperature and time, quadratically related to the frying temperature, and connected to the interaction effects of the frying temperatures and time. Hence, the predictive models presented in Table 2 could be satisfactorily employed in predicting the properties of the fried falafel judging from R^2^ > 0.80 with no lack of fit [28,37,43]. Furthermore, the properties of the falafel as affected by the frying temperature and time could be predicted using the data charted in Figure 3.

### 3.4. Optimum Frying Conditions Determination

**Figure 4:**
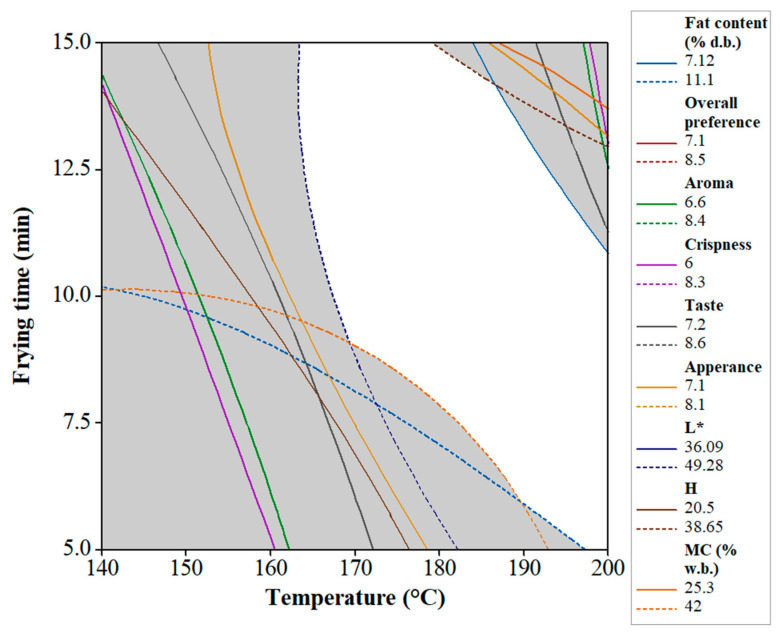
Overlaid contour plots of MC (w.b. %), hardness (N), FC (d.b. %), L*-value, appearance, crispness, taste, aroma, and overall preference of fried falafel as a function of frying temperature and time.

The suitable optimum conditions of the food frying process have a crucial role in preventing overcooking that could accordingly affect the food quality. Hence, overlaying the contour plots of the responses was used to determine the optimal frying conditions. Chambers and Wolf [44] reported that the consumer’s decision is affected by the overall preference of the products. Thus, mainly, the overall preference scores can be used to determine the optimum area. In this case, an overall preference score of at least 6 (like) was selected as the minimum value for consumer satisfaction. To achieve the optimum frying region, limitations such as the overall impression score ≥ 6, 36.1 ≤ L* ≤ 49.2, 25.3% ≤ moisture content ≤ 42 %, 20.5 N ≤ hardness ≤ 38.6 N, 7.2 % ≤ fat content ≤ 11.1 were taken into consideration. The optimum region (white shaded area) resulted from superimposing and the contour plots of the above limitations can be observed in Figure 4. Therefore, the predicted optimum conditions of T = 178.8 °C and t = 11.1 min would be used in preparing the optimum fried falafel corresponding to the above boundaries. Table 3 shows the predicted responses of the properties (FC, MC, hardness, color, and overall preference) obtained by the software.

### 3.5. Comparison among Characeristics of Deep-Fat and Air-Fried Falafel

**Figure 5:**
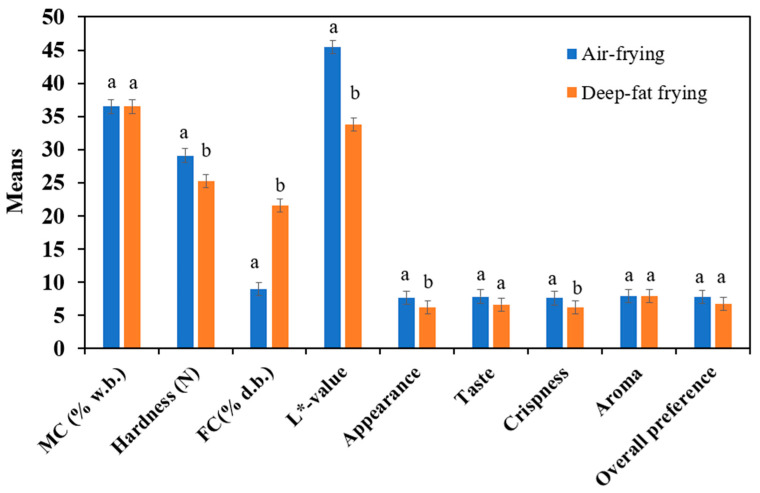
Comparison among different attributes of optimal hot air and deep fat-fried falafel obtained. Different letters indicate the significant difference. While similar letters indicate non-significant difference.

The results of physicochemical and quality attributes of the falafel, affected by air frying and control (deep-oil frying), were compared. It was found that the deep-fat fried falafel had the same moisture content as the air-fried falafel at optimal conditions (Figure 5). This result could be attributed to the fact that the moisture loss in food products results from the increase in temperature.

The hardness of food products is negatively linked to their moisture content, where the hardness of the product increases as the moisture content decreases [28]. Figure 5 revealed that the hardness values of the air-fried falafel and deep-fat fried falafel were 29.12 and 25.22 N, respectively. The highest hardness value of the air-fried falafel indicated a higher breaking force needed to fracture the fried falafel leading to more crispiness. However, both the air-fried and deep-fat fried falafel had a crispy crust and soft crumb. Similar outcomes are found for fried potato [2] and French fries [5].

Noticeably, the fat content of the optimal air-fried falafel reduced by around 45% after being fried at 178 °C for 11 min. Nevertheless, an opposite trend was detected for the deep-fried falafel as the fat content increased by 32.8% after being fried for 7 min at 180 °C. This could be good evidence that air-fried foods are lower in fat content compared to the foods cooked by the traditional deep-fat frying method. This result is in agreement with that reported by Abd Rahman et al. [2]. It was suggested that numerous factors might affect the complication of deep-fat frying mechanisms such as moisture content of food products, frying period, frying temperature, oil characteristics, the product structure, chemical reactions, and the drainage time [5]. These results indicated that the air frying technology is a good choice for frying the falafel product, especially for health-oriented people.

On the other hand, it can be seen from Figure 5 that the L* value of air-fried falafel was greater than that for deep-fat fried falafel, meaning that the deep-fat falafel is darker than the air-fried falafel. This change in color could be attributed to the water loss and oil absorbed as a result of heat and mass transfer [1]. This outcome agreed with that previously reported for several products such as tofu [45], chicken nuggets [46], and sweet potato snack [2].

A comparison between the air-fried falafel and deep-fat fried falafel of the sensory attributes is shown in Figure 5. The statistical outputs revealed that there were significant increases in appearance and crispiness scores of the optimal air-fried falafel. Although the taste and overall preference scores of the air-fried falafel were higher than those for deep-oil fried falafel, the statistical outcome revealed that the increases in taste and overall preference scores were not significant. In addition, both air-fried and deep-fat fried falafel showed similar aroma scores.

## 4. Conclusions

In this investigation, changes in the quality traits of falafel—influenced by the frying conditions using the full factorial experimental design—were comprehensively examined. The findings showed that the frying temperature and time considerably affected the moisture content, hardness, fat content, L*-value, sensory appearance, aroma, taste, crispness, and overall preference of the fried falafel. Additionally, second-order polynomial equations were established for predicting the attributes of the fried falafel in relation to the frying temperature and time. The optimum frying temperature and time were represented using contour plots to produce a fried falafel with good sensory characteristics. Accordingly, the preferable traits of the fried falafel could be achieved using the optimal frying conditions (178.8 °C and 11 min). In comparison, the air-fried falafel was lower in fat content, higher in hardness with more acceptable appearance and crispness scores than deep-fat fried falafel. Finally, the current outcomes could be advantageous for developing analytical gauges to control the quality of fried falafel throughout the frying process in the food industry.

## Figures and Tables

**Table 1:** Experimental design including the factors of frying process and their levels.

Run.	$\mathrm{Temperature},\;x_{1}\;({}^{\circ}C)$		$\mathrm{Time},\;x_{2}\;(\min)$	
	Coded	Actual	Coded	Actual
1	−1	140	−1	5
2	−1	140	0	10
3	−1	140	1	15
4	0	170	−1	5
5	0	170	0	10
6	0	170	1	15
7	1	200	−1	5
8	1	200	0	10
9	1	200	1	15

**Table 2:** Statistical constants of the second-degree polynomial equation for the quality and sensory properties of the air-fried falafel.

Property	Statistical Constants of The Second-Degree Model $y_{n}=\beta_{0}+\beta_{1}x_{1}+\beta_{2}x_{2}+\beta_{12}x_{1}x_{2}+\beta_{11}x_{1}{}^{2}+\beta_{22}x_{2}{}^{2}\;\;$							
	$\beta_{0}$	$\beta_{1}$	$\beta_{2}$	$\beta_{12}$	$\beta_{11}$	$\beta_{22}$	Lack of Fit	R^2^
MC (%w.b)	7.40	0.738 *	−1.379 *	0.0068 *	−0.0028 *	−0.0871 *	0.507	0.979
Hardness (N)	−76.90 *	0.932 *	−2.40 *	0.0114 *	−0.0022 *	0.1057 *	0.058	0.955
FC (%d.b)	3.61	0.215 *	−0.83 *	−0.000978	−0.000782 *	0.02471 *	0.422	0.965
L^*^-value	46.1 *	0.412 *	−1.276 *	−0.0028	−0.001945 *	0.0611 *	0.557	0.976
Sensory appearance	−33.67 *	0.3549 *	1.56 *	−0.0067 *	−0.0008 *	−0.0178 *	0.133	0.929
Sensory aroma	−64.12 *	0.7307 *	1.383 *	−0.0073 *	−0.0019 *	−0.0020	0.487	0.934
Sensory taste	−55.16 *	0.6094 *	1.511 *	−0.0077 *	−0.0015 *	−0.0060	0.087	0.910
Crispness	−74.09 *	0.8399 *	1.456 *	−0.0073 *	−0.0022 *	−0.0049	0.514	0.931
Overall preference	−55.26 *	0.6094 *	1.511 *	−0.0077 *	−0.0015 *	−0.0060 *	0.087	0.910

* Refers to that the effect is significant at *p*-value ≤ 0.05.

**Table 3:** Predicted responses for the properties of air-fried falafel.

Response	Fit	SE Fit *	95% CI **
MC (% w.b.)	36.48	0.548	(35.34; 37.62)
Hardness (N)	29.12	0.918	(27.21; 31.03)
FC (% d.b.)	8.99	0.201	(8.55; 9.38)
L*-value	45.46	0.465	(44.49; 46.43)
Appearance	7.61	0.129	(7.34; 7.88)
Taste	7.86	0.176	(7.49; 8.22)
Crispiness	7.61	0.16	(7.28; 7.95)
Aroma	7.89	0.146	(7.58; 8.19)
Overall preference	7.76	0.176	(7.39; 8.12)

Signs * and ** represent standard error of fit and confidence interval, respectively.

## Data Availability

The data that support the findings of this study are available from the corresponding author upon reasonable request.
